# Long-term effects of cerebral hypoperfusion on neural density and function using misery perfusion animal model

**DOI:** 10.1038/srep25072

**Published:** 2016-04-27

**Authors:** Asuka Nishino, Yosuke Tajima, Hiroyuki Takuwa, Kazuto Masamoto, Junko Taniguchi, Hidekatsu Wakizaka, Daisuke Kokuryo, Takuya Urushihata, Ichio Aoki, Iwao Kanno, Yutaka Tomita, Norihiro Suzuki, Yoko Ikoma, Hiroshi Ito

**Affiliations:** 1 Biophysics Program, Molecular Imaging Center, National Institute of Radiological Sciences, 4-9-1 Anagawa, Chiba 263-8555, Japan; 2 Department of Neurosurgery, Kimitsu Chuo Hospital, 1010 Sakurai, Kisarazu, Chiba 292-8535, Japan; 3 Brain Science Inspired Life Support Research Center, University of Electro-Communications, 1-5-1 Chofugaoka, Chofu, Tokyo 182-8585, Japan; 4 Diagnostic Imaging Program, Molecular Imaging Center, National Institute of Radiological Sciences, 4-9-1 Anagawa, Inage-ku, Chiba 263-8555, Japan; 5 Department of Neurology, Keio University School of Medicine, 35 Shinanomachi Shinjuku-ku, Tokyo 160-8582, Japan; 6 Advanced Clinical Research Center, Fukushima Global Medical Science Center, Fukushima Medical University, 1 Hikariga-oka, Fukushima 960-1295, Japan

## Abstract

We investigated the chronic effects of cerebral hypoperfusion on neuronal density and functional hyperemia using our misery perfusion mouse model under unilateral common carotid artery occlusion (UCCAO). Neuronal density evaluated 28 days after UCCAO using [^11^C]flumazenil-PET and histology indicated no neurologic deficit in the hippocampus and neocortex. CBF response to sensory stimulation was assessed using laser-Doppler flowmetry. Percentage changes in CBF response of the ipsilateral hemisphere to UCCAO were 18.4 ± 3.0%, 6.9 ± 2.8%, 6.8 ± 2.3% and 4.9 ± 2.4% before, and 7, 14 and 28 days after UCCAO, respectively. Statistical significance was found at 7, 14 and 28 days after UCCAO (*P* < 0.01). Contrary to our previous finding (Tajima *et al.* 2014) showing recovered CBF response to hypercapnia on 28 days after UCCAO using the same model, functional hyperemia was sustained and became worse 28 days after UCCAO.

Cerebral blood flow (CBF) is regulated to maintain the energy supply and metabolism in a normal state. When cerebral perfusion pressure (CPP) is decreased by circulatory disease, cerebral vasodilation is caused by the mechanism of cerebral autoregulation in order to compensate for the decrease in CBF^1^. After the lower limit of autoregulation, CBF gradually decreases and the oxygen extraction fraction increases to maintain normal oxygen metabolism and neuronal function. The situation of the brain is known as misery perfusion^2^, which has been reported to be a predictor of subsequent stroke in both medically and surgically treated patients with symptomatic major cerebral artery disease^3^. In addition, chronic cerebral hypoperfusion (CCH) has a risk of cognitive function decline and is thought to lead to neurologic deficit^4^,^5^.

Impairments of brain due to CCH have previously been investigated in animal studies. A rat model of bilateral occlusion of the common carotid arteries (BCCAO) showed a white matter lesion and a decline in working memory performance in a water maze, in spite of the absence of brain tissue atrophy, including hippocampus and striatum^6^,^7^,^8^. In the case of a mouse model of right unilateral common carotid artery occlusion (UCCAO), white matter lesion in brain and deficits in memory ability by object recognition examination were also observed^9^. We recently demonstrated a mouse model of misery perfusion resulting from permanent UCCAO^10^. A previous study reported that the percentage change in CBF response to hypercapnia was inversely correlated with cerebral vasodilation after UCCAO. The present result directly validated the measurement of CBF response to hypercapnia as being a useful assessment of the cerebrovascular reserve. Moreover, because our animal model of permanent UCCAO showed a chronic mild decrease in CBF and a decrease in the CBF response to hypercapnia, this indicated that misery perfusion occurred in our animal model^10^.

As mentioned above, previous studies have demonstrated that CCH causes white matter lesion and attenuation of cognitive function, suggesting morphological and functional damage of brain cells under misery perfusion. In the present study, we investigated whether neurological deficit, brain atrophy and reduction of functional hyperemia occurred in the mouse model of misery perfusion. At first, to estimate neurological deficit and brain atrophy, [^11^C]flumazenil ([^11^C]FMZ) positron emission tomography (PET) and histology were performed with our mouse model. [^11^C]FMZ is a ligand of the central benzodiazepine receptor, which is a component of the ubiquitous GABA_A_ complex and was commonly used as a PET marker for neuronal (GABA_A_) density in both human and animal models^11^,^12^,^13^,^14^,^15^. Second, we investigated the long-term effect of misery perfusion on functional hyperemia, which represents a cellular communication among neurons, glias, and vascular cells^16^. To evaluate functional hyperemia, the CBF response to sensory stimulation was measured using laser-Doppler flowmetry (LDF). Our results showed selective impairment of functional hyperemia, associated with CCH in the animal model of misery perfusion.

## Results

### Baseline of CBF after UCCAO

**Figure 1:**
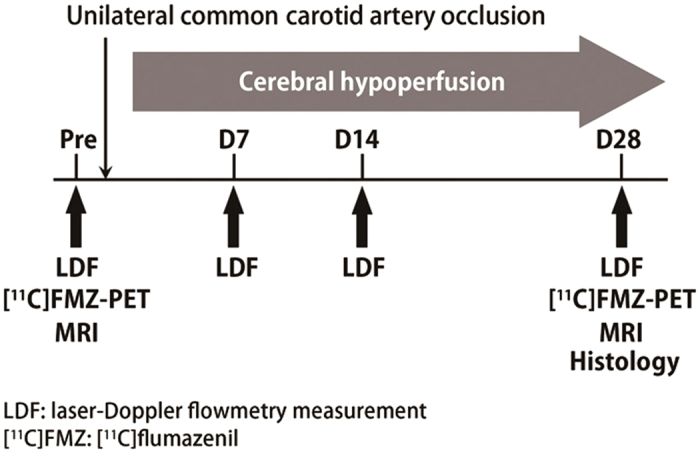
Experimental protocol for [^11^C]FMZ-PET, histology, MRI and LDF measurement. LDF measurements were performed before and at 7, 14 and 28 days after UCCAO from both contralateral and ipsilaeteral sides in awake mice. CBF response was evoked by whisker stimulation (10 Hz, 20-sec) and percentage change in CBF was calculated offline. [^11^C]FMZ-PET and MRI were performed before and 28 days after UCCAO. Histology was performed at 28 days after UCCAO.

**Figure 2:**
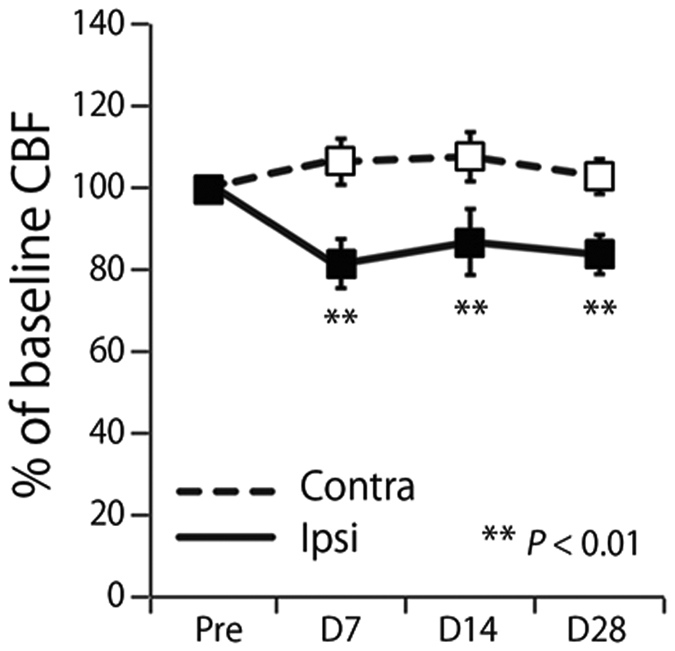
Comparison between before and after UCCAO of the baseline CBF during resting state. Solid and dashed lines show baseline CBF of the ipsilateral and contralateral hemispheres to UCCAO, respectively. Asterisks show significant difference between contralateral and ipsilateral sides. Error bars represent SD.

Experimental protocol shows in Fig. 1. Baseline CBF of the ipsilateral cerebral hemisphere to UCCAO decreased 82.4 ± 2.2%, 86.5% ± 4.6%, and 83.2% ± 3.6% of the preoperative value at 7, 14, and 28 days after UCCAO, respectively (Fig. 2). Statistically significant differences were found at 7, 14, and 28 days (*P* < 0.01) after UCCAO as compared with the preoperative value. Meanwhile, in the baseline CBF of the contralateral cerebral hemisphere, there were no statistically significant differences throughout the experimental period.

### Estimation of neural density before and 28 days after UCCAO with [^11^C]FMZ-PET

**Figure 3:**
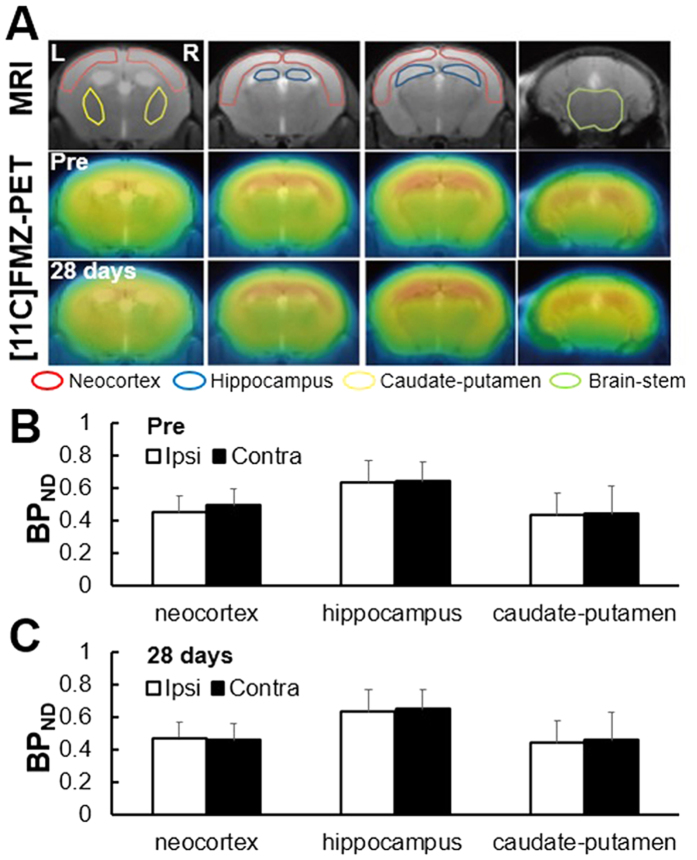
(**A**) Representative transaxial brain images in structural MRI and [^11^C]FMZ-PET 28 days after UCCAO. The top images are MRI. The middle and bottom images are PET before and at 28 days after UCCAO, respectively. (**B**) BP_ND_ values of neocortex, hippocampus and caudate-putamen before UCCAO. Black and white bars indicate ipsilateral and contralateral side, respectively. (**C**) BP_ND_ values of neocortex, hippocampus and caudate-putamen 28 days after UCCAO.

Figure 3A shows the representative brain images of MRI and [^11^C]FMZ-PET at 1 month after UCCAO. Accumulation of [^11^C]FMZ was observed in the whole brain, including neocortex, hippocampus and caudate-putamen (Fig. 3A). Before UCCAO, the binding potential (BP_ND_) values of neocortex in the contralateral and ipsilateral hemispheres to UCCAO were 0.45 ± 0.10 and 0.49 ± 0.10, respectively, those in hippocampus were 0.63 ± 0.14 and 0.64 ± 0.12, respectively, and those in caudate-putamen were 0.43 ± 0.14 and 0.44 ± 0.17, respectively (Fig. 3B). There were no significant differences in BP_ND_ of these three brain areas between the contralateral and ipsilateral hemispheres before UCCAO. Twenty-eight days after UCCAO, the BP_ND_ values of neocortex in the contralateral and ipsilateral hemispheres to UCCAO were 0.47 ± 0.12 and 0.46 ± 0.14, respectively, those in the hippocampus were 0.63 ± 0.15 and 0.65 ± 0.12, respectively, and those in the caudate-putamen were 0.44 ± 0.14 and 0.46 ± 0.10, respectively (Fig. 3C). There were no significant differences in BP_ND_ of these three brain areas between the contralateral and ipsilateral hemispheres 28 days after UCCAO.

**Figure 4:**
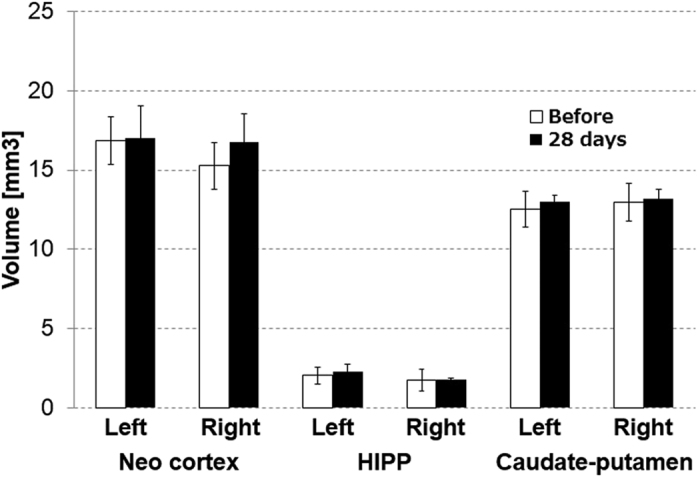
Volumes of neocortex, hippocampus and caudate-putamen before and 28 days after UCCAO. Black and white bars indicate volumes before and 28 days after UCCAO, respectively.

T2-weighted MR images showed that our occlusion model developed no detectable infarction in brains (Fig. 3A), which was in good agreement with the previous results of Tajima *et al*.^10^. Before UCCAO, the volumes of the neocortex in the contralateral and ipsilateral hemispheres were 16.9 ± 1.5 mm^3^ and 15.2 ± 1.5 mm^3^, respectively, and those in the hippocampus were 2.1 ± 0.5 mm^3^ and 1.8 ± 0.7 mm^3^, respectively, and those in caudate-putamen were 12.6 ± 1.26 and 13.0 ± 1.36, respectively (Fig. 4). There were no significant differences in the volume of these three brain areas between the contralateral and ipsilateral hemispheres before UCCAO. Twenty-eight days after UCCAO, the volumes of the neocortex in the contralateral and ipsilateral hemispheres were 17.0 ± 2.1 mm^3^ and 16.7 ± 1.8 mm^3^, respectively, and those in the hippocampus were 2.3 ± 0.5 mm^3^ and 1.8 ± 0.11 mm^3^, respectively and those in caudate-putamen were 13.0 ± 0.19 and 13.2 ± 0.38, respectively (Fig. 4). There were no significant differences in the volumes of these three brain areas between the contralateral and ipsilateral hemispheres 28 days after UCCAO.

### Immunohistological assessment of brain tissue at 28 days after UCCAO

**Figure 5:**
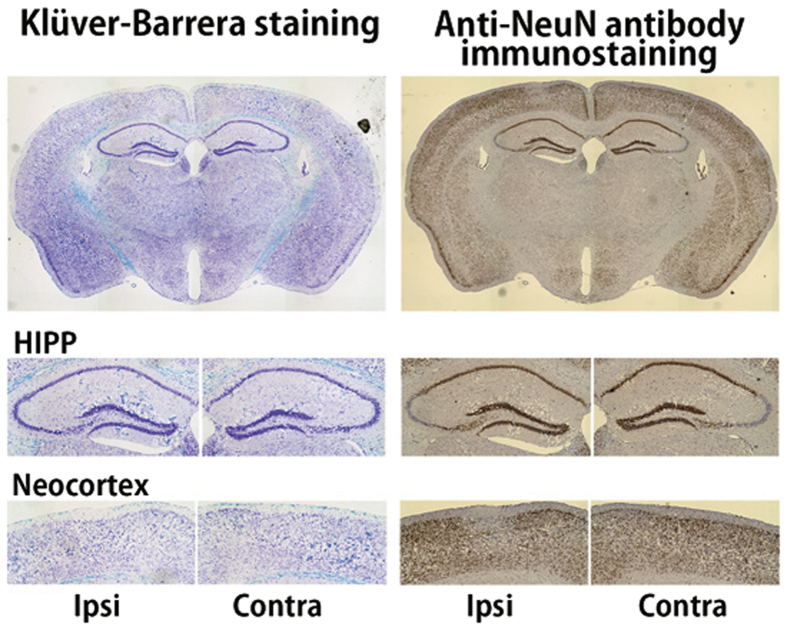
Immunohistochemical assessment of brain tissue 28 days after UCCAO. Top, middle and bottom images show whole brain, hippocampus and neocortex area, respectively. Left samples are stained with Klüver-Barrera, and right samples with anti-NeuN.

Figure 5 shows a representative paraformaldehyde (PFA)-fixed sample of cerebral slice stained by Klüver-Barrera (KB) method and anti-neuronal nuclear antibody (NeuN) immunostaining from a mouse 28 days after UCCAO. KB method is a Luxol fast blue (for myelin; blue color) combined with cresyl violet stain (for neurons; purple color). Anti-NeuN is a nucleoprotein marker, with which the depth of the color density shows normal neurons. The quantity of staining of both sides of the neocortex and hippocampus were at the same level, and the survival of neurons was confirmed regardless of UCCAO.

### Effects of chronic hypoperfusion on CBF response to sensory stimulation

**Figure 6:**
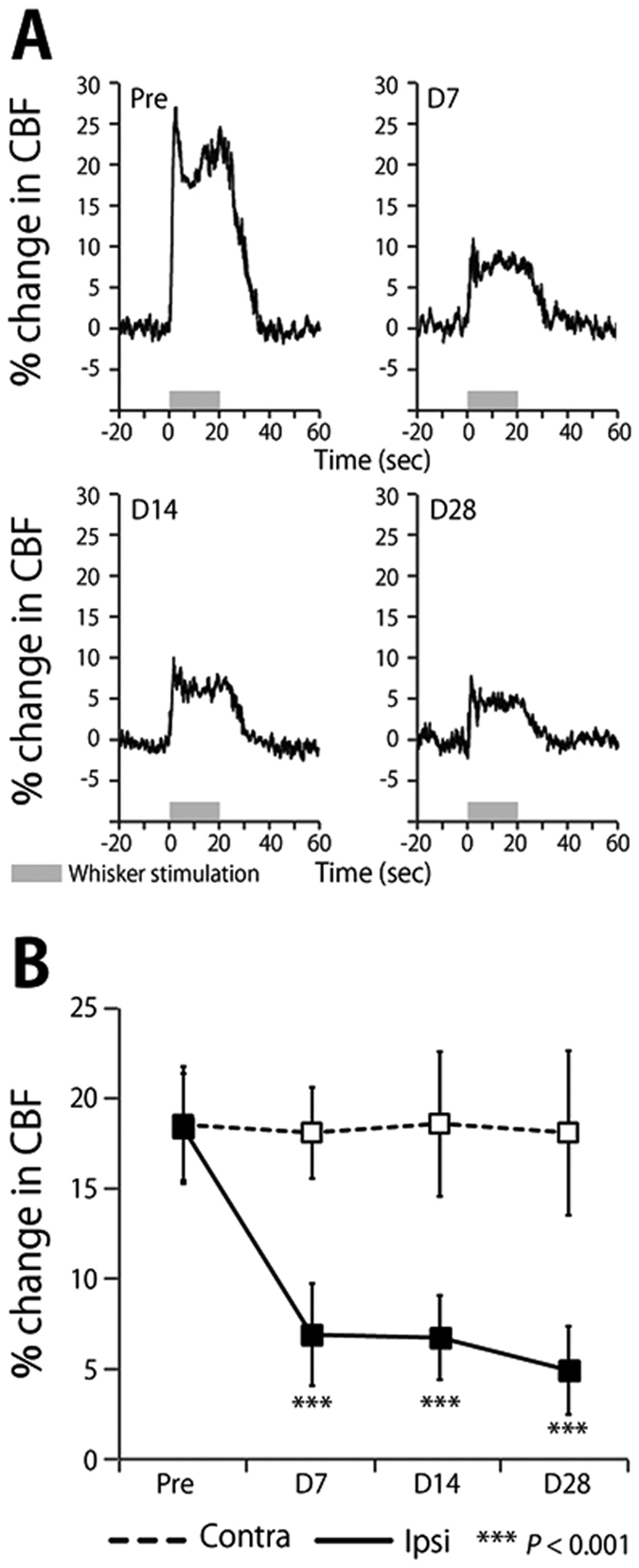
(**A**) Time–response curves of the increase in CBF response to sensory stimulation before and at 7, 14 and 28 days after UCCAO. Horizontal gray bars indicate the sensory stimulation period. (**B**) Mean percentage change during sensory stimulation measured from the barrel cortex at contralateral and ipsilateral sides of UCCAO. Error bars represent SD.

A representative time-response curve of CBF increase during sensory stimulation in the ipsilateral hemisphere to UCCAO is shown in Fig. 6A. Time–response curves of CBF showed that both signals exhibited an initial peak followed by a plateau during sensory stimulation, then declining slowly to the baseline level. Attenuation in CBF increase during sensory stimulation was observed after UCCAO. The percentage changes in CBF of the contralateral hemisphere to UCCAO were 18.6 ± 3.2%, 18.1 ± 2.5%, 18.6 ± 4.0% and 18.1 ± 4.6% before, and at 7, 14 and 28 days after UCCAO, respectively (Fig. 6B). The percentage changes in CBF of the ipsilateral hemisphere to UCCAO were 18.4 ± 3.0%, 6.9 ± 2.8%, 6.8 ± 2.3% and 4.9 ± 2.4% before, and at 7, 14 and 28 days after UCCAO, respectively (Fig. 5B). Statistically significant differences were found at 7, 14 and 28 days after UCCAO (*P* < 0.001).

## Discussion

CCH leads to a decline of cognitive functions in animals subjected to either UCCAO or BCCAO^8^,^9^, indicating that CCH-triggered cognitive dysfunction is closely associated with neurologic deficits or neural dysfunctions. However, our results of [^11^C]FMZ-PET, structural MRI, and immunohistochemistry examinations all consistently showed that neither neurologic deficit nor brain atrophy was observed in the neocortex and hippocampus after one month of CCH (Figs 3, 4, 5), which was quite contrary to our expectation. Importantly, we found that the sustained reduction of functional hyperemia in this phase of CCH occurred only in the ipsilateral side of UCCAO (Fig. 6). We thought that ameliorating the functional hyperemia could be a potent therapeutic target against neurologic deficits elicited by CCH.

The sustained reduction of the functional hyperemia observed during CCH could be due to an impairment of vascular functions and/or a breakdown of neurovascular interfaces because of the preserved neural properties in this early phase of CCH (Figs 3 and 4)^10^. previously demonstrated a significant increase in the diameter of the cerebral artery from 1 to 28 days after the induction of UCCAO, and that the CBF response to hypercapnia initially decreased and then significantly recovered from 14 days after UCCAO. In the present study, we observed a sustained reduction of the CBF response to sensory stimulation after the induction of UCCAO (Fig. 6), which differed from the previous observation of the CBF response to hypercapnia during CCH^10^. This discrepancy could indicate that CCH impairs the neurovascular coupling not via vascular dysfunction but rather a breakdown of the neurovascular interfaces in our experimental conditions. This notion seems to agree well with our previous observation of the hypoxia-induced adaptation of the functional hyperemia^17^,^18^. In that study, we found that CBF response to sensory stimulation was selectively reduced after exposure to chronic hypoxia in the mouse cortex, despite normally preserved neural activities and vascular responses to hypercapnia^17^. Whether cerebral hypoxia is associated with this CCH model should be determined in future works.

It has been shown that a mouse model of CCH expresses white matter lesion^7^,^9^, including glial activation preferentially evoked in white matter, but less in gray matter^19^. Consistent with these histological observations^20^, demonstrated that brain cells in *in vivo* mouse cortex were maintained intact morphologically under CCH. Furthermore, later development of hippocampal atrophy and cognitive impairment in CCH mouse, three months after the induction of UCCAO, was recently noted^21^. Considering these observations, we speculate that sustained reduction of functional hyperemia caused by CCH leads to a later development of neurologic deficits, including brain atrophy and selective neuronal loss^22^,^23^. Further follow-up studies over a prolonged period of CCH with [^11^C]FMZ-PET would be necessary to elucidate the causal relationship between the impaired neurovascular unit and neurologic deficits developed during CCH.

## Materials and Methods

### Animal preparation

Three separate measurements were performed using 8 male C57BL/6J mice (20–30 g, 8–10 weeks old; Japan SLC, Inc, Hamamatsu) in total: 1) [^11^C]FMZ-PET experiments (n = 6), 2) LDF experiments (n = 6), 3) immunohistochemical method (n = 2). [^11^C]FMZ-PET and LDF experiments were performed using the same animals. Two additional animals were used in the immunohistochemistry experiments. All mice were housed individually in separate cages with water and food *ad libitum*. Mouse cages were kept at a temperature of 25 °C in a 12-h light/dark cycle.

All animal experiments were approved by the Institutional Animal Care and Use Committee of the National Institute of Radiological Sciences (Inage, Chiba, Japan) and were performed in accordance with the institutional guidelines on humane care and use of laboratory animals approved by the Institutional Committee for Animal Experimentation.

During the surgical preparation for LDF, a mixture of air, oxygen, and isoflurane (3–5% for induction and 1.5–2% for surgery) anesthesia was given via face-mask. The mouse heads were fixed with a stereotactic frame, and two cranial windows were prepared according to the ‘Seylaz-Tomita method’^24^. The cranial windows were attached over the left and right sides of the somatosensory cortex using dental cement (Luxaflow, DMG, Hamburg, Germany), centered at 1.8 mm caudal and 2.5 mm lateral from the bregma. A custom-made metal plate was affixed to the front of the central skull. The detailed method for preparing the chronic cranial window was reported in previous studies^10^,^24^,^25^. All experiments were performed 2 weeks after the cranial window surgery.

For the UCCAO surgical procedure, a mixture of air, oxygen, and isoflurane (3–5% for induction and 1.5–2% for surgery) anesthesia was given by face mask. A midline cervical incision was made and the right common carotid artery was isolated from the adjacent vagus nerve and double-ligated using 6–0 silk sutures.

LDF experiments were repeatedly performed before, and at 7, 14 and 28 days after UCCAO surgery. [^11^C]FMZ-PET and histology were conducted before and 28 days after UCCAO.

### [^11^C]FMZ-PET studies

PET scans to evaluate the density of nerve cells were performed with a small-animal PET (Inveon; Siemens Medical Solutions USA, Knoxville, TN, USA) after the LDF study (Fig. 1). Mice were anesthetized with isoflurane (3% for induction and 1.5% for maintenance) via face mask, and rectal temperature (37˚C) was maintained using a heat therapy pump (T/PUMP, GAYMAR, Orchard Park, NY, USA) and incandescent lamp.

A bolus of 30–37 MBq of [^11^C]FMZ was administered from the tail vein by 31-gauge needle by catheter, and dynamic scan in 3D list mode was acquired for 60 min after the injection. List mode data were rebound as 20 time frames (1 min × 4, 2 min × 8, 5 min × 8) and reconstructed with filtered back projection using a Hanning filter with a Nyquist cutoff of 0.5 cycle/pixel.

Regions of interest (ROIs) were manually drawn on the individual MR images for the neocortex, hippocampus, and brain stem. PET images were coregistered to the corresponding MR images, and the respective time-activity curves (TACs) of [^11^C]FMZ were extracted from the dynamic PET images. In each TAC, the area under the curve (AUC) was calculated by integrating the tissue radioactivity concentration from 30 to 60 min as an estimate of the cumulative radioactivity concentration of [^11^C]FMZ in the latter part of the scanning. The BP_ND_ of [^11^C]FMZ was expressed as follows:


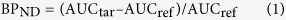


where AUC_tar_ is the AUC in the target region (neocortex, hippocampus and caudate-putamen) and AUC_ref_ is the AUC in the reference region (brain stem)^26^. Statistical analysis was performed to compare [^11^C]FMZ BP_ND_ between before and 1 month after UCCAO using paired t-test. All data analyses of [^11^C]FMZ-PET were carried out using PMOD software (PMOD Technologies Ltd., Zurich, Switzerland).

### Histology and immunohistochemistry examinations

Mouse brains were removed and fixed with 4% PFA. After initial fixation in PFA, brain tissue was sliced into 10-μm-thick coronal sections using conventional techniques. For myelin and nerve cells, the sections were stained with KB method for histology. Immunohistochemical staining of the brain sections was performed according to the avidin–biotin complex method, using primary anti-NeuN (ab177487, Abcam, Cambridge, UK). After section deparaffinization with xylene and gradual dehydration, endogenous peroxidase activity was blocked with 0.5% hydrogen peroxide (H_2_O_2_) for 15 min. They were incubated with 10% normal goat serum (G9023; Sigma–Aldrich) in phosphate-buffered saline (PBS) with diluted primary antibody (1:2000) overnight at 4 °C. Then they were washed in PBS containing 0.05% Tween-20 (PBST), incubated overnight at 4 °C with biotinylated goat anti-rabbit IgG antibody (BA-1000; Vector Labs, Burlingame, CA, USA; 1:1000) as secondary antibody, and washed in PBST again. After incubation with Vectastain ABC Reagents (PK-6100; Vector Labs; 1:1000) for 2 h, the sections were washed in PBST, and finally, staining was visualized by reaction with 3,3-diaminobenzidine tetrahydrochloride (Sigma–Aldrich) and 0.03% H_2_O_2_ in Tris-buffered saline for 10 min. Stained sections were examined under a light microscope.

### Laser-Doppler flowmetry experiments

Measurement of CBF was performed using an LDF system (FLO-C1, OMEGAWAVE, Tokyo, Japan). The tip of the LDF probe (0.46-mm diameter; Type NS, OMEGAWAVE) was placed perpendicular to the cerebral cortex with a guide tube, which was attached to the cranial window on the barrel cortex that avoided large blood vessels^19^. The experimental protocol for CBF measurement by LDF system in awake mice was reported previously^25^. The time course of the LDF signal was recorded with an analog data recorder system (MP150, BIOPAC Systems, Goleta, CA, USA) at a sampling rate of 200 Hz, which was analyzed offline.

CBF response during neuronal activation was evoked by whisker stimulation in awake state. An air-puff was delivered to mouse whiskers on the contralateral side of the LDF measurement at a pressure of ~15 psi by compressed-air bottle. A rectangular pulse stimulation of 50-ms pulse width and 100-ms onset-to-onset interval (i.e., 10-Hz frequency) generated by Master-8 (A.M.P.I., Jerusalem, Israel) was induced for a 20-s duration^25^. In each experiment, 15 consecutive trials were repeated with an onset-to-onset interval of 120 s. The LDF signal was normalized by 20-s pre-stimulus baseline level, and averaged across 15 trials. The magnitude of evoked CBF was calculated as the mean percentage change relative to baseline. Baseline CBF and the CBF response to sensory stimulation were statistically analyzed across different experimental days using one-way analysis of variance with repeated measures, followed by Tukey’s test. Statistical significance was assumed for values of *P* < 0.05.

### Magnetic Resonance Imaging Experiments

MRI experiments to monitor the brain condition (e.g. infarction and atrophy) after UCCAO were performed with a 7.0 T horizontal MRI scanner (Magnet: Kobelco and JASTEC, Kobe, Japan; Console: Bruker Biospin, Ettlingen, Germany), with a volume coil for transmission (Bruker Biospin) and a 2-channel phased array surface coil for reception (Rapid Biomedical, Rimpar, Germany). The mice were initially anesthetized with 3.0% isoflurane (Escain, Mylan Japan, Tokyo, Japan) and then with 1.5% to 2.0% isoflurane and 1:5 oxygen/room-air mixture during the MRI experiments. Rectal temperature was continuously monitored using an optical fiber thermometer (FOT-M, FISO, Quebec, QC, Canada) and maintained at 37.0 °C ± 0.5 °C using a heating pad (Temperature control unit, Rapid Biomedical), and warm air was provided by a homemade automatic heating system based on an electric temperature controller (E5CN, Omron, Kyoto, Japan) throughout the MRI experiments. During MRI scanning, the mice lay in a prone position on an MRI-compatible cradle and were held in place by handmade ear bars. The first imaging slices were carefully set at the rhinal fissure, with reference to a mouse brain atlas.

Transaxial T2-weighted images before and 28 days after UCCAO were acquired using rapid acquisition with a relaxation enhancement (RARE) sequence as follows: TR/effective TE = 4,200/36 ms, slice thickness = 1.0 mm, slice gap = 0.0 mm, Fat-Sup = on, matrix = 256 × 256, FOV = 25.6 × 25.6 mm^2^, number of acquisitions = 4, RARE factor = 8, number of slices = 13, scan time = 6 min 43 s. The volumes of the neocortex and hippocampus were measured before and at 28 days after the treatment using ImageJ (NIH).

## Additional Information

**How to cite this article**: Nishino, A. *et al.* Long-term effects of cerebral hypoperfusion on neural density and function using misery perfusion animal model. *Sci. Rep.* **6**, 25072; doi: 10.1038/srep25072 (2016).
